# Painless Dentistry

**Published:** 1898-07

**Authors:** Clyde Payne

**Affiliations:** San Francisco.


ARTICLE VI.

### PAINLESS DENTISTRY.

DR. CLYDE PAYNE, SAN FRANCISCO.

We owe it to our patients to use the means at our hands to make the operations painless. There is no operation in the mouth that cannot be made quite painless. It takes a little more time, but the patient is usually willing to pay for the extra time.

Regarding the formula I use—carboonate of potassium, glycerine cocaine and carbolic acid in a saturated solution—I have had excellent results with it, as have Drs. Younger, Cool, Lewis and others who have used it. It is the next best thing for sensitive dentine to cataphoric medication. Apply the rubber-dam, dry out the cavity thoroughly—dry it out with alcohol; place a drop of the obtundent into the cavity and throw a continuous blast of hot air on it, and keep it up for five minutes, when you can excavate quite painlessly.

The basis of nearly all local anesthetics is cocaine. It s a dangerous drug to use if you do not understand its physiological action; but for hypodermic injections for the painless extraction of teeth, I use the following formula, in which the ingredients are so proportioned that I am yet o have an ill-physiological effect from cocaine : Cocaine, 15 grains; glycerine, 5 drams; nitro-glycerine, 1-10 of a grain; sulphates of morphia and atropia, 1 grain; carbolic acid, 3 drops; and distilled water sufficient to make a two-ounce mixture. There is sufficient glycerine to localize the cocaine holding it in apposition to the parts a sufficient length of time to complete the operation, and not very long so that it acts as an irritant and causes a swelling. In patients who have a poor circulation sometimes there is a swelling with this formula, but it will be painless and will subside as soon as the anesthetic, with which you have infiltrated the tissues, has become absorbed. The nitro-glycerine stimulates the heart just in proportion as the cocaine may depress it. The sulphates of morphia and atropia overcome the after-pain. The carbolic acid keeps the solution.

I will give you an idea of the sterility of this combination. Dr. S. E. Knowles used some that had been standing nine months with a perfect result. The anesthetic figures a 1½ per cent. solution of cocaine.

Many of you are using this formula. I mention it again for the benefit of those who might wish to try it. This is the first time that the formula has been made public property, though I gave it to every one that asked me for it.

Operations of implantation can be made with this formula without pain. Dr. W. J. Younger removed a tumor from the mouth of a patient after I had anesthetized the parts, without pain. The wound healed by first intention.

I had occasion to assist Dr. W. A. Bryant in an operation where surgical means were taken to correct a very pronounced superior protrusion. The four incisors were extracted, four new sockets drilled and the teeth replanted. The operation was painless.

I use it always before adjusting a clamp if the clamp is to impinge on the gum.—*Dental Brief.*

